# Selective ultrasound contrast enhancement in the tumor by nanocapsules with perfluorooctylbromide: effect of PLGA–PEG proportion

**DOI:** 10.1039/c8ra01824c

**Published:** 2018-05-16

**Authors:** Zheng Wang, Jinsong Ding, Xiaoqian Ma, Shengjuan Luo

**Affiliations:** Department of Hepatobiliary Surgery, The Third Xiangya Hospital, Central South University Changsha Hunan 410013 PR China; School of Pharmaceutical Sciences, Central South University Changsha Hunan 410013 PR China; Department of Radiology, The Third Xiangya Hospital, Central South University Changsha Hunan 410013 PR China; Department of Ultrasound, The Third Xiangya Hospital, Central South University Changsha Hunan 410013 PR China sameluo@csu.edu.cn

## Abstract

We used PLGA–COOH and PLGA–PEG–COOH blended polymer material to encapsulate perfluorooctyl bromide to prepare nanocapsules (NCs) as nano-ultrasound contrast agents. The aim of this study was to assess the effect of PLGA–PEG proportion on the physical, biological and acoustic characteristics of the nanocapsules, and to develop optimal nanocapsules for selective ultrasound contrast enhancement in tumors. The weight ratio of PLGA–PEG in the formulation was 0, 25%, 50%, 75%, and 100%, and the corresponding nanocapsules were designated NCs_PLGA_, NCs_25% PLGA–PEG,_ NCs_50% PLGA–PEG,_ NCs_75% PLGA–PEG_ and NCs_100% PLGA–PEG_. As the PLGA–PEG proportion increased, the diameter and bulk modulus of the NCs gradually decreased, and the originally smooth surface of NCs was roughened. NCs_PLGA_, NCs_25% PLGA–PEG_ and NCs_50% PLGA–PEG_ had regular spherical shape and relatively distinct boundaries compared with NCs_75% PLGA–PEG_ and NCs_100% PLGA–PEG_, which showed heavy agglomeration. The proportion of PLGA–PEG in the formula could also change the uptake rate of NCs by RAW 264.7 cells. NCs_50% PLGA–PEG_ and NCs_75% PLGA–PEG_ had the lowest uptake by RAW 264.7 cells. *In vitro*, the ultrasonic gray values of NCs_50% PLGA–PEG_, NCs_75% PLGA–PEG_ and NCs_100% PLGA–PEG_ were obviously higher than those of NCs_PLGA_ and NCs_100% PLGA–PEG_. NCs_PLGA_, NCs_50% PLGA–PEG_ and NCs_100% PLGA–PEG_ were injected into mice *via* the tail vein, but only NCs_50% PLGA–PEG_ could produce persistent gray contrast enhancement in tumors after 24 h. Histological fluorescence of the tumor tissue confirmed that NCs_50% PLGA–PEG_ and NCs_100% PLGA–PEG_ gathered in tumor tissues. Our results indicate that the PLGA–PEG proportion in the formula is an important factor in constructing optimal nano-ultrasound contrast agents with a liquid core, and could change the nanocapsule size, surface morphology, elastic modulus, macrophage cellular uptake, and ultrasonic reflection. An appropriate PLGA–PEG proportion could help nanoparticles to achieve selective gray contrast enhancement in tumors.

## Abstract 2

Effects of different PLGA–PEG proportion on nanoparticles' morphology, elastic modulus, mononuclear cell uptake rate and ultrasound contrast enhancement.

## Graphical abstract

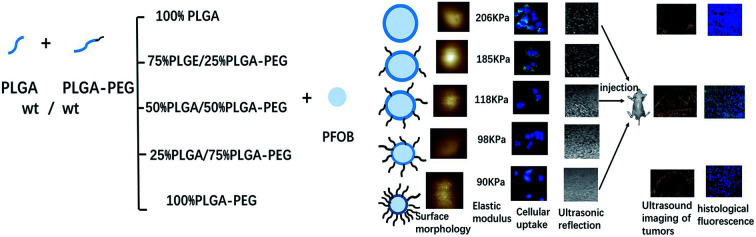

## Introduction

Liquid perfluorocarbons (PFCs) have been used as ultrasound contrast agents (UCAs) for more than 30 years since Mattrey *et al.* showed that perfluorooctylbromide (PFOB) caused persistent enhancement of the ultrasound signal in rabbit liver.^1^ PFOB is stable and has low toxicity. It is the most suitable liquid perfluorocarbon to be used *in vivo*.^2^ However, its nano-preparations have not succeeded in the implementation of selective ultrasound contrast enhancement in the tumor after intravenous injection.^3,4^ Usually, lipid or polymeric shell encapsulated PFOB is used to prepare nanoscale ultrasound contrast agents. The composition of the shell, an important factor, can change mechanical strength, elasticity and viscosity of ultrasound contrast agents to strongly affect their acoustic characteristics.^5–7^ Poly(lactic-*co*-glycolic acid) (PLGA) forms one of the most suitable contrast agent shells because of its good biodegradability and biocompatibility.^8^ However, when nanocapsules (NCs) with PLGA hydrophobic shells enter the body, they activate complement (a kind of plasma protein, called opsonin). The monocyte phagocytic system (MPS) was able to identify active complement in plasma and produce phagocytosis. NCs were cleared by the MPS before reaching the targeted organs.^9,10^ At present, the most effective and widely used method employs the non-ionized polymer polyethylene glycol (PEG) to cover the nanoparticle surface.^8^ These PEG chains are highly hydrophilic to shield hydrophobicity. PEG coverage density may change the surface properties and pharmacokinetics of the nanoparticles, binding of plasma protein *etc.*^11,12^ However, the effect of different PEG proportions in the shell on the NCs' biophysics and acoustics, and the NCs' ability for selective contrast enhancement have not been investigated so far.

Here we used a strategy in which PLGA–PEG partially or entirely replaced PLGA in the formula, and adopted a modified emulsification–solvent evaporation technique to obtain five different NCs (NCs_PLGA_, NCs_25% PLGA–PEG_, NCs_50% PLGA–PEG_, NCs_75% PLGA–PEG_, NCs_100% PLGA–PEG_) with a PFOB core.^13^ Atomic force microscopy (AFM) was used to measure NC surface topography and elastic modulus separately without multi-parameter uncertainty involved in the process.^5,14^ We also evaluated the NCs' physical and biological properties, and acoustic reflection. We injected the three contrast agents NCs_PLGA_, NCs_50% PLGA–PEG_ and NCs_100% PLGA–PEG_ into animals to compare their ultrasound contrast enhancement in tumors.

In this study, we validated that the proportion of PLGA–PEG could affect NC size, zeta potential, surface morphology and elastic modulus as well as the uptake rate of monocytes. When the PLGA–PEG proportion was appropriate, NCs with a PFOB core could be used as an ultrasound contrast agent to produce ultrasonic contrast enhancement in tumors.

## Materials and methods

### Nanocapsules preparation

Nanocapsules composed of PFOB (Aladdin, Shanghai, PR China) packaged in membrane materials PLGA (50 : 50, *M*_w_ = 18 000) and PLGA–PEG3400 (Daigang, Jinan, PR China) were synthesized using previously described methods.^3,15^ Briefly, 50 mg PLGA was dissolved in 2 ml methylene chloride along with 30 μl PFOB. The organic solution was placed in a thermostated bath at 20 °C to ensure even dispersion of the PFOB. It was then emulsified into 10 ml 1.0% polyvinyl alcohol (PVA; *M*_w_ 13 000–23 000, Sigma, St Louis, USA) aqueous solution to form a pre-emulsion. The pre-emulsion was sonicated at 300 W for 2 min over ice and then evaporated for 3 h in a thermostated bath (30 °C) to remove the methylene chloride. Then, a small amount of precipitate and large particles were removed by low speed centrifugation. After that, the solution was filtered through 0.45 μm water system membrane, and nanocapsules were obtained. There were 5 different PLGA–PEG weight ratios in the formula of the membrane material, respectively 0, 25%, 50%, 75%, and 100%. Five nanocapsules were prepared, respectively: NCs_PLGA_, NCs_25% PLGA–PEG_, NCs_50% PLGA–PEG_, NCs_75% PLGA–PEG_ and NCs_100% PLGA–PEG_. Coumarin-6 (Sigma, St Louis, USA) was added to the organic solution prior to emulsification to label the polymer.

### Measurement of NC characteristics

NC particle diameter, size distribution, and zeta potential were measured by a dynamic light scattering system (Zetasizer Nano-ZS; Malvern Instruments, Worcestershire, UK) at 25 °C.

### Encapsulation efficiency (EE)

The PFOB concentration of the supernatant liquor was measured by gas chromatography (GC) at 300 °C, using a flame ionization detector (FID). Calculating percentage encapsulation efficiency was based on the following equation.1
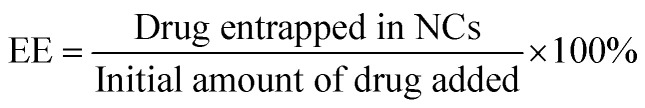


### AFM measurements

The elastic modulus and morphology were investigated by means of a Dimension Icon atomic force microscope system (AFM, Bruker, Santa Barbara, CA, USA). Images were collected by means of commercial silicon nitride probes (SNL-A, Bruker) and a Stargate scanner (max. scan size ≈ 100 μm) at 1.0 Hz scan rate. This mode was based on peak force tapping, which produced a very fast force curve at every pixel in the image by modulating the *Z* piezo at ∼1 kHz with amplitude 200 nm. Analysis of force curve data was done in real time, providing a map of multiple mechanical properties, such as modulus and adhesion, which had similar resolution to the height image.

### Interaction of nanocapsules with RAW 264.7 cells

Cellular uptake of NCs was observed by using an inverted fluorescence microscope TEZ000-S (Nikon, Tokyo, Japan) and flow cytometry (FCM; Beckman Coulter, CA, USA) using coumarin-6 (Cou-6) as the fluorescent probe as described previously. RAW 264.7 cells (Cell Institute of the Chinese Academy of Sciences, Shanghai, PR China) in Dulbecco's modified Eagle's medium (DMEM) with 10% serum were plated in 24-well plates to adhere overnight. The next day, the medium was removed, and 150 μl suspension of medium containing Cou-6-labeled NCs with different PLGA–PEG percentages was added for 2 h at 37 °C. Then cells were washed three times with phosphate-buffered saline (PBS). Some were stained with 1 mg ml^−1^ 4′,6-diamidino-2-phenylindole (DAPI) at room temperature for 20 min away from light for fluorescence microscopy and others for flow cytometry.

### *In vitro* ultrasound imaging

Each NC suspension sample was placed in an Eppendorf tube. Ultrasound images were obtained in nonlinear mode with a commercial ultrasound imaging system (L74 clinical ultrasound probe, Hitachi, Japan). All images were acquired using the same instrument parameters: frame rate (FR) 26, brightness (BG) 20, and dynamic range (DR) 65 db.

### Tumor-bearing mouse model

Approximately 1.0 × 10^7^ HepG2 cells (Cell Institute of the Chinese Academy of Sciences, Shanghai, PR China) were inoculated subcutaneously into the right hind legs of BALB/c mice (5 weeks old; Jingda Experimental Animal Co. Ltd, Changsha, PR China). All *in vivo* experiments began when the tumors reached a diameter of 0.8–1.2 cm.

### *In vivo* ultrasound imaging

The mice were anesthetized with 1% pentobarbital sodium by abdominal injection (1 mg/100 g); 0.3 ml samples were injected *via* the tail vein. After injection, we recording the images of the tumor continuously for 10 minutes and then after 0.5 h, 2 h, 12 h, 24 h. No instrument parameters were changed during this experiment.

### Histological analysis

NCs_PLGA_, NCs_50% PLGA–PEG_ and NCs_100% PLGA–PEG_ (0.3 ml) labeled with coumarin-6 were respectively injected into tumor-bearing mice (*via* the tail vein). There were three mice in each group. Twenty-four hours after being injected, the mice were killed, and tumors were collected and sectioned into 5 μm slices. Frozen sections were stained with DAPI. Images were obtained by means of a fluorescence microscope (Olympus BX51, Japan). DAPI and coumarin-6 were excited at 340 and 466 nm, respectively, and the emission was recorded at 488 and 504 nm, respectively.

### Statistical methods

All experiments were conducted in triplicate. All data were expressed as the mean ± SD. Statistical analyses were performed by using one-way analysis of variance (ANOVA). All experiments were in accordance with the requirements of the relevant ethical guidelines (such those of the International Association for the Study of Pain). IACUC approved the experiments to be conducted in the Animal Experimental Center.

## Results

### Characteristics of nanocapsules

Characteristics of nanocapsules are shown in Table 1.

**Table 1:** Size distribution, zeta potential and encapsulation efficiency (EE) for different NC formulations

NC formulation	PEG (% w/w)	Size (nm)	PDI	Zeta potential (mV)	% EE
PLGA	0	255.4 ± 16	0.17	−23.59 ± 3.2	60.14 ± 6.57
25% PLGA–PEG	4.7	236.6 ± 16	0.23	−11.25 ± 3.0	69.50 ± 3.81
50% PLGA–PEG	9.4	225.0 ± 6	0.24	−10.30 ± 2.6	66.64 ± 8.10
75% PLGA–PEG	14.2	201.3 ± 20	0.29	−7.64 ± 2.7	58.77 ± 5.84
100% PLGA–PEG	18.9	171.6 ± 17	0.25	−6.36 ± 1.8	64.24 ± 6.88

Particle size was between 170 and 255 nm with small polydispersity index (PDI). Zeta potential values were greatly reduced when PLGA–PEG was added into the membrane material (*P* < 0.05). No significant difference of the zeta potential values was found for PLGA–PEG percentages ranging from 25% to 100%. The encapsulation efficiency results showed no difference among the groups (*P* > 0.05).

### Surface morphology and elastic modulus by AFM

**Fig. 1:**
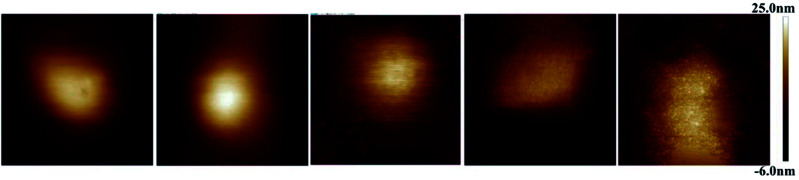
From left to right, surface morphology images of NCs_PLGA_, NCs_25% PLGA–PEG_, NCs_50% PLGA–PEG_, NCs_75% PLGA–PEG_ and NCs_100% PLGA–PEG_.

Fig. 1 displays AFM surface morphology images of NCs with different PLGA–PEG percentages. The surface of NCs_PLGA_ was smooth. With the increase of PLGA–PEG percentage, the smooth surface gradually became uneven. When PLGA–PEG completely replaced PLGA, the surface was the roughest. We also found that NCs_PLGA_, NCs_25% PLGA–PEG_ and NCs_50% PLGA–PEG_ were of regular spherical shape with relatively distinct boundaries. But for NCs_75% PLGA–PEG_ and NCs_100% PLGA–PEG_, heavy agglomeration occurred.

**Fig. 2:**
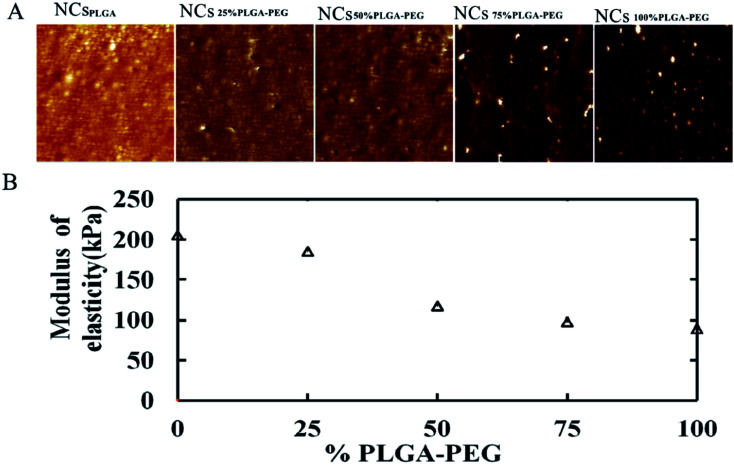
Elastic modulus of NCs_PLGA_, NCs_25% PLGA–PEG_, NCs_50% PLGA–PEG,_ NCs_75% PLGA–PEG_ and NCs_100% PLGA–PEG_ in suspension. (A) Images of the elastic modulus of NCs, with the weight percentage of PLGA–PEG gradually increasing (0, 25, 50, 75, 100%) in the formulation. (B) Plot of the corresponding nanocapsules' bulk modulus as a function of PLGA–PEG percentage.

Fig. 2A shows elastic modulus images of NCs with PEG at different percentages. When the PLGA–PEG percentage increased from 0 to 100, the bulk modulus of nanocapsules decreased from 205.67 to 89.7 MPa (Fig. 2B).

### Cellular uptake studies

**Fig. 3:**
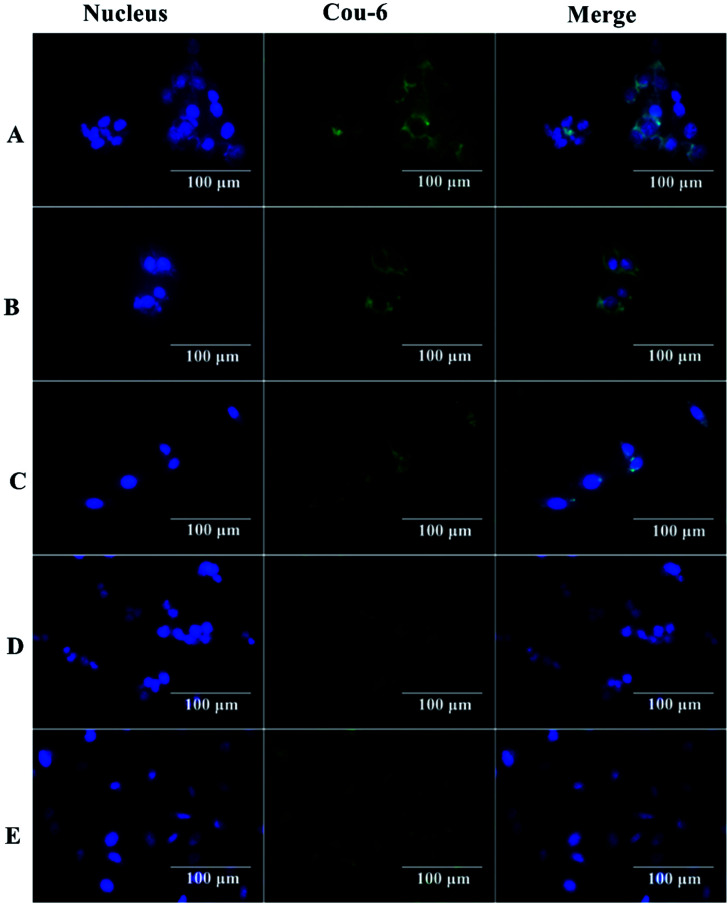
Fluorescence images of RAW 264.7 cells after incubation with Cou-6-loaded NCs. (A) NCs_PLGA_, (B) NCs_25% PLGA–PEG_, (C) NCs_50% PLGA–PEG_, (D) NCs_75% PLGA–PEG_, (E) NCs_100% PLGA–PEG_. The blue shows nuclei stained by DAPI. The green shows NCs loaded with Cou-6.

**Fig. 4:**
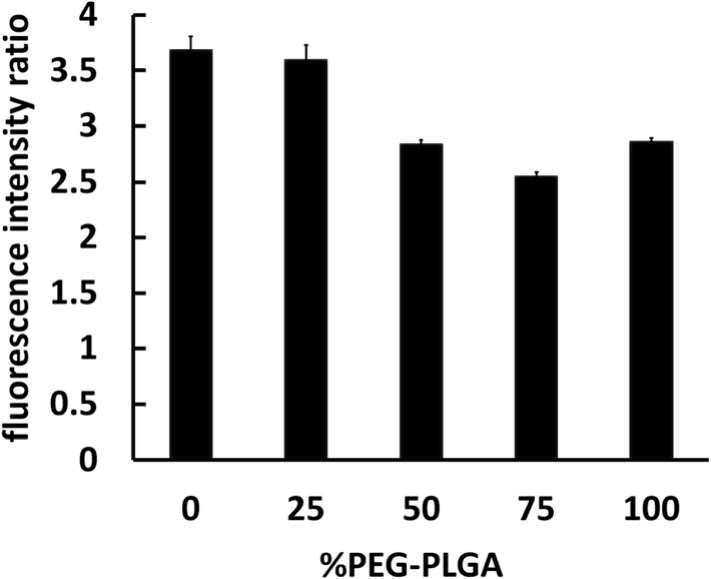
Fluorimetric quantitative analysis of RAW 264.7 cells interacting with different NCs loaded with Cou-6.

The interaction between nanocapsules labeled with coumarin-6 and macrophage-like RAW 264.7 cells could be observed by fluorescence microscopy. The studies showed that more intense green fluorescence was observed in cells exposed to NCs_PLGA_ and NCs_25% PLGA–PEG_ (Fig. 3). The actual amount of internalized Cou-6-labeled NCs was investigated by flow cytometry. Higher uptake was observed for NCs_PLGA_ and NCs_25% PLGA–PE_, and lower uptake for NCs_50% PLGA–PEG_, NCs_75% PLGA–PEG_ and NCs_100% PLGA–PEG_ (*P* ≤ 0.000). Compared with NCs_100% PLGA–PEG_, the uptake percentage of NCs_50% PLGA–PEG_ and NCs_75% PLGA–PEG_ was lower (*P* = 0.036, *P* = 0.013, Fig. 4).

### *In vitro* ultrasonic reflection of different NCs

**Fig. 5:**
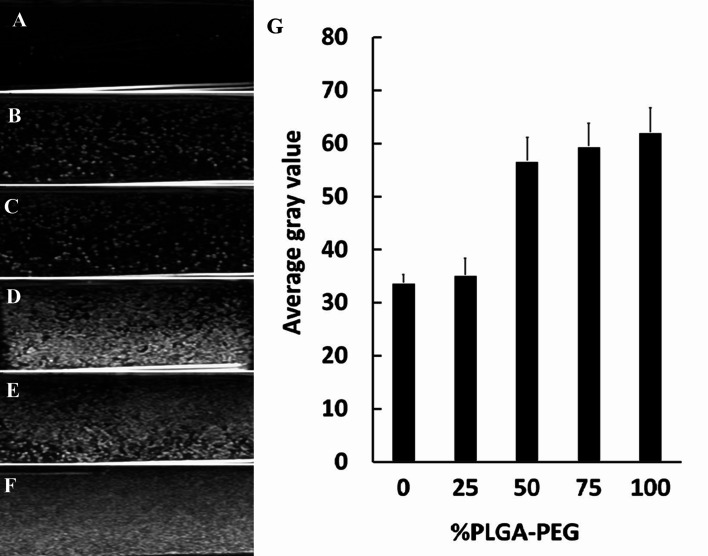
*In vitro* ultrasound images of different nanocapsule suspensions. (A) Degassed deionized water (control), (B) NCs_PLGA_, (C) NCs_25% PLGA–PEG_, (D) NCs_50% PLGA–PEG_, (E) NCs_75% PLGA–PEG_, (F) NCs_100% PLGA–PEG_. (G) Quantitative analysis of echo-signal intensity with different NCs.

To assess the native echogenicity of NCs with PLGA–PEG at different percentages, the gray enhancement of the ultrasound image was measured. The ultrasound images of degassed deionized water (used as a control) and NCs with PLGA–PEG at different percentages are shown in Fig. 5. Compared with water (Fig. 5A), the tested NCs appeared obviously brighter (Fig. 5B–F). The ultrasonic reflection of NCs_50% PLGA–PEG_, NCs_75% PLGA–PEG_ and NCs_100% PLGA–PEG_ was significantly stronger than that of NCs_PLGA_ and NCs_25% PLGA–PEG_. Ultrasonic signal reflection intensity was quantified, and the mean gray scales corresponding to the NCs are shown in Fig. 5G. The signal intensity was increased significantly for PLGA–PEG 50%, 75%, and 100% (*P* = 0.000), compared with PLGA, and slowly increased with the increasing PLGA–PEG content (*P* > 0.05).

### Ultrasound-enhanced imaging of tumors

**Fig. 6:**
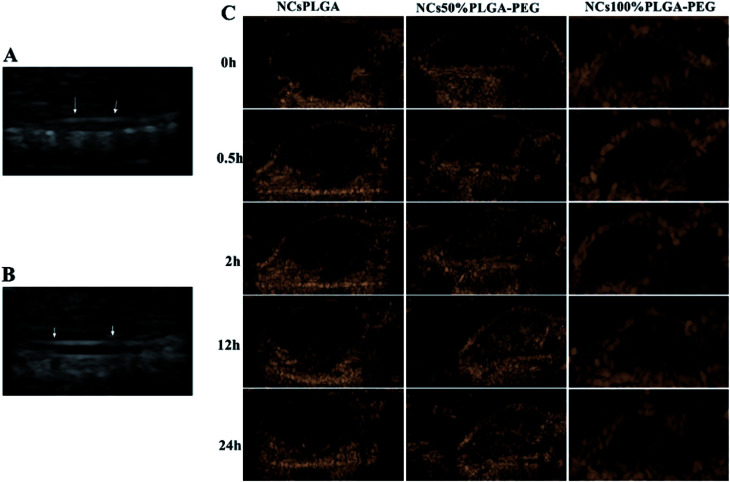
The inferior vena cava (indicated by arrows) presents significant enhancement after NC injection (A), and appears dark before injection (B). (C) *In vivo* ultrasonic imaging of tumor-burdened mice before and after the injection of NCs.

**Fig. 7:**
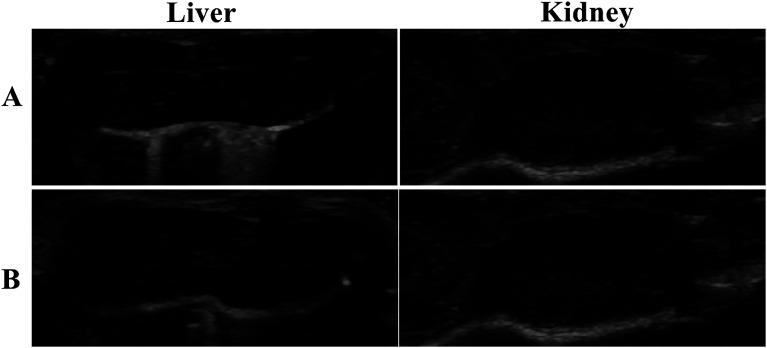
Ultrasound images of liver and kidney before (A) and after (B) injection of NCs.

Tumor-bearing nude mice were divided into three groups at random: NCs_PLGA_ group (4 mice), NCs_50% PLGA–PEG_ group (4 mice), NCs_100% PLGA–PEG_ group (4 mice). After injection, gray contrast enhancement was observed at once in the inferior vena cava, which lasted for several seconds in the three groups (Fig. 6A and B). The gray contrast enhancement was observed in the NCs_50% PLGA–PEG_ group 2 h after injection, and continued for 24 h (Fig. 6C). Before and after injection, tumors appeared dark in the NCs_PLGA_ and NCs_100% PLGA–PEG_ groups. In addition, the images in liver and kidney were also observed before and after injection. The results showed no significant gray contrast enhancement in the three groups (Fig. 7).

### Histological fluorescence

**Fig. 8:**
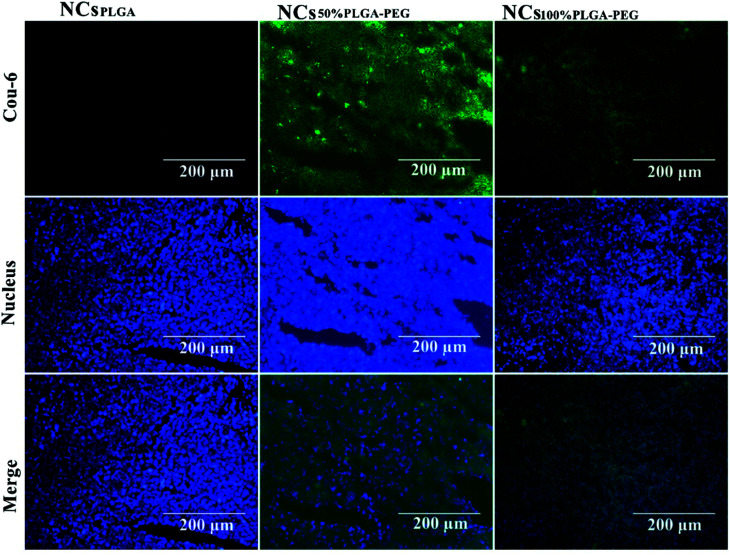
Histological fluoroscopy images of frozen sections after nuclear labeling. Many green NCs_50% PLGA–PEG_ and NCs_100% PLGA–PEG_ labeled with coumarin-6 were observed in the tumor intercellular space; NCs_PLGA_ labeled with coumarin-6 were hardly detected in the tumor intercellular space. Blue represents nuclear staining.

Fig. 8 shows typical histological fluorescence images of tumor slices from mice injected with NCs_PLGA_, NCs_50% PLGA–PEG_ or NCs_100% PLGA–PEG_. Tumor tissue of the nucleus was dyed blue by DAPI. Green fluorescence was not observed for tumors of mice injected with NCs_PLGA_. By contrast, green fluorescence signals were observed from the tumor tissue of mice injected with NCs_50% PLGA–PEG_ or NCs_100% PLGA–PEG_. Bright green spots appeared outside the nucleus in the superposition image.

## Discussion

Nanoparticle-sized liquid perfluorocarbon was bound to a specific targeted surface to increase ultrasonic reflectivity so as to produce ultrasonic contrast enhancement, in a different manner from microbubble agents, which depend on harmonic resonance to enhance ultrasonic backscatter.^16,17^ The shell played an important role in the biophysics and echogenicity of the liquid perfluorocarbon contrast agent.^15,18^ In this laboratory study, we assessed the shell's biophysical and acoustic impact on NCs by using a strategy in which PLGA–PEG partially or entirely replaced PLGA in the formulation to modify the NC shell.

We clearly noticed a trend of decreasing particle size when the percentage of PLGA–PEG increased. A similar finding was obtained with rhodamine-loaded PEG-*g*-PLA.^19^ It was probably due to the amphiphilic nature of PEG–PLGA copolymers reducing the interfacial tension between the aqueous and organic phases. Compared with the PLGA group, zeta potential decrease in the PLGA–PEG groups gave indirect evidence that the PEG moieties were located at the surface of the NCs.^20^ Zeta potential values varied a little in different PLGA–PEG groups. This could also be explained by residual PVA on the NP surfaces, reducing the actual zeta potential values.^19^ The percentage of PEG–PLGA in the shell did not influence the encapsulation (*P* > 0.05). Indeed, the hydrophilic characteristic of PEG kept it directed towards the aqueous phase, while the hydrophobic core of PLGA could entrap the hydrophobic drugs. Hydrophilic PEG was directly exposed to the aqueous phase, and the hydrophobic PLGA formed a solid shell to package the PFOB.

AFM provided simultaneous, high-resolution imaging and mechanical property mapping of nanoparticles.^21,22^ In contrast to electron microscopy, AFM does not require dry samples or use of a vacuum, but can be used directly for samples in aqueous solution, which is a great advantage.^7,22^ In Fig. 1 (from A to D), the smooth surface of the nanocapsules gradually became rough, which directly indicated the existence of PEG chains at the surface of the nanocapsules.^23^ The elastic modulus of particles could extremely influence circulation lifetime and biodistribution.^24^ The softer the particles were, generally the longer they circulated *in vivo*, and the more easily they avoided immune system phagocytosis and passed through narrow micro-channels.^25,26^ The particles' elastic modulus could also affect cellular internalization and trafficking of nanoparticles.^27^ PEG coverage-density is important in achieving avoidance of the monocyte phagocytic system (MPS). Usually, high surface coverage by PEG chains could evade the MPS.^19^ In this experiment, when the PLGA–PEG proportion was ≥50%, the uptake of NCs by macrophage cells was less.

In a previous study by Sanna *et al.*, PLGA-*b*-PEG-based microbubbles produced more echo reflection than PLGA.^18^ From mathematical analysis of ultrasound Rayleigh scattering from liquid-filled polymeric nanocapsules, Flegg *et al.* concluded that the shell played an important role in the scattering from each nanocapsule, and therefore also played a role in the echogenicity of an agglomeration.^28^ In our current work, we found that the proportion of PLGA–PEG could affect the scattering of ultrasonic waves by nanocapsules.

Ultrasound contrast enhancement was observed after NCs_50% PLGA–PEG_ was injected into tumor-bearing mice. Fluorescence microscopy confirmed the presence and the accumulation of NCs_50% PLGA–PEG_ in the tumor tissue. This accumulation, resulting from passive targeting of the nanocapsules, was attributed to the stealthiness of the PEGylated surface, and the tumor vascular permeability. When enough NCs_50% PLGA–PEG_ gathered in the tumors, the ultrasonic reflection could significantly increase, and produce ultrasonic gray enhancement visible by eye. The accumulation of NCs_100% PLGA–PEG_ was detected in tumor tissue, but no ultrasound enhancement contrast was observed in the tumors, probably because the amount of NCs_100% PLGA–PEG_ was not sufficient for ultrasonic imaging. NCs_PLGA_, lacking PEG, were destroyed by the MPS, so they did not show up in the tumor tissue.

## Conclusion

In this research, PLGA and PLGA–PEG blended copolymers were used to prepare nanocapsule-enclosed PFOB. When the PLGA–PEG proportion was changed in the formulation, the size, zeta potential, surface morphology and elastic modulus of the NCs changed, and their biological properties, mainly macrophage cellular uptake, and acoustic responses changed as well. We confirmed NCs could produce ultrasound contrast enhancement in tumors after intravenous injection. The results suggested that the nanoscale ultrasound contrast agent encapsulated PFOB prepared by optimizing the PLGA–PEG ratio in membrane materials successfully achieved selective contrast enhancement of nanoparticles in tumors.

## Conflicts of interest

The authors report no conflicts of interest.

## Supplementary Material
